# Anti-parasitic effect of vitamin C alone and in combination with benznidazole against *Trypanosoma cruzi*

**DOI:** 10.1371/journal.pntd.0006764

**Published:** 2018-09-21

**Authors:** Vanesa Puente, Agostina Demaria, Fernanda M. Frank, Alcira Batlle, Maria Elisa Lombardo

**Affiliations:** 1 Centro de Investigaciones sobre Porfirinas y Porfirias, CIPYP (UBA-CONICET), Hospital de Clínicas José de San Martín, UBA, Buenos Aires, Argentina; 2 Instituto de Microbiología y Parasitología Médica, IMPAM (Universidad de Buenos Aires—Consejo Nacional de Investigaciones Científicas y Técnicas), Facultad de Medicina, UBA, Buenos Aires, Argentina; 3 Cátedra de Inmunología, Facultad de Farmacia y Bioquímica, UBA, Buenos Aires, Argentina; 4 Departamento de Química Biológica, Facultad de Ciencias Exactas y Naturales, UBA, Buenos Aires, Argentina; Ohio State University, UNITED STATES

## Abstract

**Background:**

Drugs currently used for the treatment of Chagas’ disease, nifurtimox and benznidazole, have a limited effectiveness and toxic side effects. With the aim of finding new therapeutic approaches, *in vitro* and *in vivo* anti-*Trypanosoma cruzi* activity of vitamin C alone and combined with benznidazole were investigated.

**Methodology/Principal findings:**

The trypanocidal activity on epimastigote and trypomastigote forms was evaluated by counting parasites in a Neubauer chamber after treatment with the compounds. For the amastigote stage, transgenic parasites expressing β-galactosidase were used and quantified by measuring the β-galactosidase activity. The cytotoxicity of compounds was tested on Vero cells. The redox state of the parasite was evaluated by determining the reduced thiol levels (spectrophotometric assay) and the intracellular oxidative state (by flow cytometry). The *in vivo* trypanocidal activity was evaluated on a murine model of Chagas’ disease. The trypanocidal activity of vitamin C and benznidazole was similar for the three parasite forms. When combining both drugs, vitamin C did not induce any change in the antiparasitic activity of benznidazole on trypomastigotes; however, on mammal cells, vitamin C diminished the cytotoxicity degree of benznidazole. Two mechanisms of action may be postulated for vitamin C: a lethal pro-oxidant effect on the parasite when used alone, and an antioxidant effect, when combined with benznidazole. A similar behavior was observed on infected mice; i.e., parasite counts in infected mice treated with vitamin C were lower than that of the control group. Animals treated with benznidazole presented lower parasitemia levels, as compared with those treated with vitamin C alone. Again, vitamin C did not cause any effect on the antiparasitic profile of benznidazole. Even though a combined treatment was employed, the antioxidant effect of vitamin C on the host was evidenced; a 100% survival was observed and the weight loss occurring during the acute phase of the infection was reduced.

**Conclusions/Significance:**

Based on these results, the combination of vitamin C with benznidazole could be considered as an alternative treatment for Chagas’ disease. These preliminary results encourage further research to improve the treatment of Chagas’ disease.

## Author summary

The huge worldwide expansion of Chagas’ disease (American trypanosomiasis) that has occurred as a result of the population mobility from Latin America, has caused this parasitic disease to become an important topic for the World Health Organization. Cases of Chagas’ disease have been reported in the United States and Canada, as well as in Europe and the Western Pacific. The efficacy of current drugs (nifurtimox and benznidazole) to treat this disease is limited. Besides, these drugs cause adverse effects. For these reasons, a continuous research to find therapeutic alternatives is required. In our laboratory, we evaluate the anti-*Trypanosoma cruzi* effect of both synthetic and natural products. Promising results have been obtained in our laboratory when the effect of vitamin B_12_ was tested on infected mice. As a continuation of that work, herein we evaluated the effects of the combined treatment of vitamin C and benznidazole as a therapeutic alternative. Vitamin C was found to diminish the cytotoxicity of the antichagasic drug.

## Introduction

*Trypanosoma cruzi* is the causative agent of Chagas’ disease, which was declared of worldwide interest by the World Health Organization (WHO) [1]. To fight this illness that originated in Latin America (American trypanosomiasis), nifurtimox and benznidazole (Bnz) are used. These drugs are known to cause considerable secondary effects and have limited effectiveness. For this reason, over the past years, studies have been undertaken to find novel therapeutic alternatives to treat this disease.

Many drugs have been reported to exert their anti-*T*. *cruzi* effect through the generation of reactive oxygen species (ROS) [2–4]. The infection with *T*. *cruzi* is known to cause and inflammatory response together with the generation of oxidative stress, which plays a central role in the pathophysiology of the disease [5–8]. Both events suggest that the production of ROS could be an efficient strategy against the parasite. It is known that ascorbic acid or vitamin C (Vit C) exerts a dual effect, acting as antioxidant at physiological concentrations (40–80 μM), and as a pro-oxidant at pharmacological high concentrations (1 to 5 mM, achieved only by intravenous administration) [9,10]. Ascorbate acts as an antioxidant neutralizing potentially harmful free radicals, while as pro-oxidant, in the presence of catalytic metallic ions, ascorbate induces the formation of H_2_O_2_ and ROS.

The pro-oxidant effect is the main mechanism by which high doses of Vit C cause cell death. Thus, Vit C can manifest its known selective cytotoxic effect on tumor cells, which are particularly vulnerable to the oxidative stress induced by H_2_O_2_ [10–12]. Such pro-oxidant effect could also account for the antiparasitic activity previously demonstrated in our laboratory when *T*. *cruzi* parasites were treated with a combination of Vitamin B_12_ and Vit C [3]. In this case, the induction of the toxic effect would be mediated by cobalt ions contained in the corrinic ring of the vitamin B_12_.

*T*. *cruzi* can either synthesize ascorbic acid or obtain it from the culture medium [13]. Our hypothesis is that Vit C has the capacity to kill the parasite acting as a pro-oxidant compound by reacting with traces of heavy metal ions present inside the parasite´s cytoplasm and/or protect the host from the oxidative stress generated by the *T*. *cruzi* infection. Thus, the antiparasitic activity of Vit C was studied *in vitro* on the three stages of *T*. *cruzi*, as well as *in vivo* using an acute Chagas’ disease murine model. The effect caused by the combined administration of Vit C and benznidazole (Bnz) was also assessed.

## Materials and methods

### Chemicals

Hemin, EDTA, NADH, RPMI-1640 culture medium, dithionitrobenzoate (DTNB) and L(+) ascorbic acid GR (Vit C) were obtained from Sigma Chem. Co. (Saint Louis, MO, USA). Yeast extract, tryptose and brain heart infusion (BHI) were from Difco Laboratories (Sparks, MD, USA). Bnz was kindly provided by Roche (Argentina). All other chemicals were of the highest purity commercially available.

### Parasites

*Trypanosoma cruzi* epimastigotes (Tulahuén strain) were grown at 28 ºC in a liquid medium containing 3.3% BHI (Difco), 0.3% tryptose (Difco), 0.3% disodium phosphate, 0.04% potassium chloride, 0.03% dextrose, and hemin (20 μg mL^-1^). After sterilization, penicillin (100 IU mL^-1^), streptomycin (100 μg mL^-1^) and 20% v/v heat-inactivated fetal calf serum (Natocor) were added. *T*. *cruzi* bloodstream trypomastigotes were obtained from infected CF1 mice by cardiac puncture at the peak of parasitemia on day 15 post-infection. Trypomastigotes were routinely maintained by infecting 21 day-old CF1 mice. *T*. *cruzi* parasites from the Tulahuén strain expressing the β-gal gene (Tul-β-Gal) were kindly provided by Dr. Buckner [14].

### Animals

Outbred CF1 male and inbred C3H/HeN female mice were nursed at the Departamento de Microbiología, Facultad de Medicina, Universidad de Buenos Aires.

### *In vitro* assays for anti-*T*. *cruzi* activity

To evaluate growth inhibition on epimastigotes, Vit C was tested at concentrations ranging from 1 to 10 μM. Bnz was used as reference drug at concentrations ranging from 2.5 to 10 μM. The percentage of inhibition (I%) and 50% inhibitory concentration (IC_50_) values were estimated by counting the parasites in a Neubauer chamber, as previously described [3].

The trypanocidal activity was also evaluated on bloodstream trypomastigotes. Parasites were cultured in the presence of 5 to 100 μM of Vit C or 0.38 to 380 μM of Bnz, under the conditions stated elsewhere [3]. Living parasites were then counted in a Neubauer chamber and the percentage of lysis (% L) and the concentration that causes 50% lysis (IC_50_) were calculated as previously reported [3].

The growth inhibition of amastigotes was determined in J774 murine macrophages cultures infected with transgenic trypomastigotes expressing β-galactosidase (Tul- β-Gal). Vit C and Bnz were assayed from 0.5 to 40 μM. Parasite counts were then determined spectrophotometrically by measuring the product of the enzymatic reaction. I% and IC_50_ values were then calculated as previously described [3].

### Cytotoxic activity

The cytotoxicity assay was carried out *in vitro*, on Vero cells (from the Departamento de Química Biológica, Facultad de Ciencias Exactas y Naturales, Universidad de Buenos Aires). Briefly, cells were seeded on a 24-well plate. Each well contained 500 μL of a cell suspension at 9x10^5^ cells mL^-1^. The plate was then incubated for 24 h at 37°C. After this period, Vit C (from 5 to 5000 μM) and Bnz (3 to 3000 μM) was added. The plate was incubated for 48 h at 37°C. After incubation, the culture medium was replaced with phosphate buffered saline PBS and MTT was added at a final concentration of 0.5 mg/ml. Cells were incubated for 1 h at 37°C and then resuspended in 500 μL of dimethyl sulfoxide (DMSO). The absorbance was read at 570 nm using an ELISA plate reader. Absorbance values of wells containing only medium and reagents were used as blank reaction. MTT assays were done in triplicate. The 50% cytotoxic concentration (CC_50_) was defined as that causing a 50% of death. The selectivity index (SI) was calculated as CC_50_/IC_50._

### Intracellular redox state assays

The intracellular redox state was measured on 4-day-growth epimastigotes treated with different concentrations of Vit C (5, 15 and 30 μM) during 2, 5, 8 and 10 h. The following assays were then carried out.

The content of thiol groups was determined by measuring the change in light absorbance (410 nm) occurring when SH-groups reduce DTNB. The quantification was carried out in cell-free extract, following the methodology described previously [15].

The intracellular oxidative stress was evaluated by flow cytometry using 2',7'-dichlorodihydrofluorescein diacetate (H_2_DCFDA) as fluorescent probe, following the methodology previously described [4]. The fluorescence intensity of the oxidized probe was measured in a Becton Dickinson FACScalibur flow cytometer. Results were analyzed using the FlowJo V10 software and the ratio Gmt/Gmc was determined, where Gmt and Gmc correspond to the geometric mean of histograms obtained with treated and untreated (control) cells, respectively.

### *In vivo* trypanocidal activity assay

Six to eight week-old C3H/HeN mice were infected with 5x10^3^ trypomastigotes through the intraperitoneal route. Seven days after the infection, mice were treated with either Vit C (1.5 mg/kg/day) or Bnz (0.75 mg/kg/day), or the combination of both. Bnz concentration used arise from S1 Fig. These drugs were resuspended in 0.05M PBS (pH 7.2). The control group was treated only with the vehicle. Drugs were administered from Monday to Friday during 2 weeks (days 7 to 12 and 15 to 20 post-infection) through the intraperitoneal route. Parasitemia levels were monitored every other day by counting parasites in a Neubauer chamber, and using 5 μl of blood diluted 1:5 in lysis buffer (0.75% NH_4_Cl, 0.2% Tris, pH 7.2). The number of dead mice was registered every day. The weight of each animal was registered between days 9 and 16 post-infection. Results were expressed as weight loss percentage considering 100% the body weight corresponding to day 9 post-infection.

### Ethics statement

Animal experiments were approved by the Institutional Committee for the Care and Use of Laboratory Animals (CICUAL) of Universidad de Buenos Aires, Facultad de Medicina, Argentina (identification no. 2943/2013). Animals received human care and were treated in accordance with guidelines established by the Animal Care and Use Committee of the Argentine Association of Specialists in Laboratory Animals (AADEALC), which were in accordance with the Guide for the Care and Use of the Laboratory Animals of the National Research Council of the National Academies [16].

### Statistical analysis

All data are expressed as means ± SEM. To calculate the IC_50_, I% or L% values were plotted against the log of drug concentration (μM) and fitted to a sigmoidal curve determined by a non-linear regression (Sigma Plot 12 software). The significance of differences was evaluated with either the Student´s *t* test or one-way analysis of variance (ANOVA) with *post-hoc* analysis using the Tukey’s test. A p< 0.05 was considered significant. Parasitemia and weight loss were analyzed using the non-parametric Mann-Whitney’s *U* test. Parasitemia was also expressed as Area Under the Curve (AUC). Survival curves were compared with a log-rank test. Results presented are representative of three to four independent experiments.

## Results

### *In vitro* antiparasitic activity

**Fig 1:**
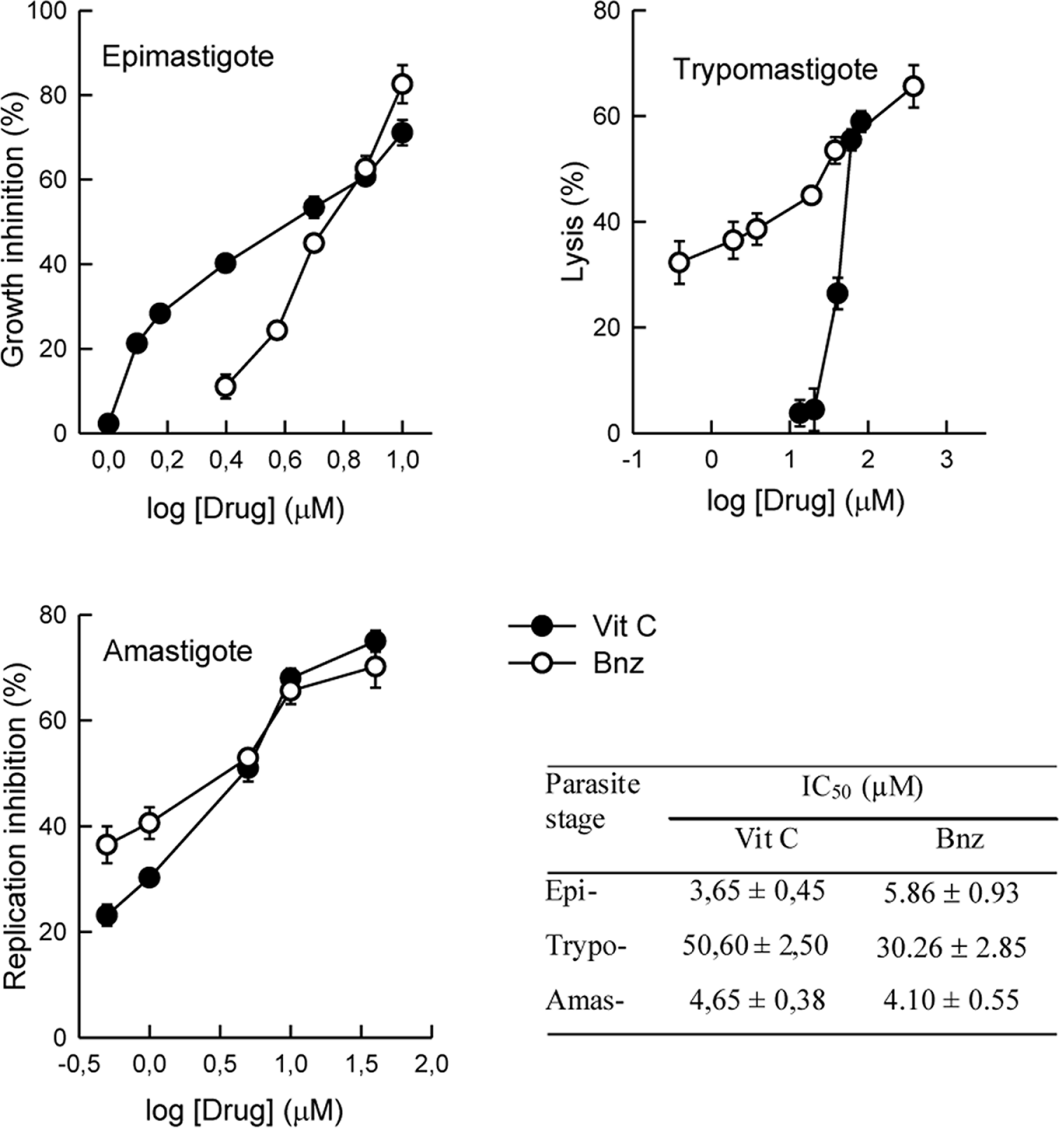
Antiparasitic activity of Vit C and Bnz on *T*. *cruzi* epimastigote, trypomastigote and amastigote forms. Vit C concentrations assayed ranged from 1 to 10 μM for epimastigotes, 5 to 100 μM for trypomastigotes and 0.5 to 40 μM for amastigotes. For Bnz, used as reference drug, the concentrations tested ranged from 2.5 to 10 μM for epimastigotes, 0.38 to 380 μM for trypomastigotes and 0.5 to 40 μM for amastigotes. IC_50_ values were calculated (see Materials and Methods) and shown in the inset table.

The *in vitro* antiparasitic activity of Vit C was evaluated on the three parasite stages (Fig 1). On epimastigotes, Vit C was found to be 1.3–1.8 times more active than Bnz, whereas Bnz was 1.4 to 1.8 times more active than Vit C on bloodstream trypomastigotes. In amastigotes, similar IC_50_ values were obtained for Vit C and Bnz.

In order to carry out an *in vivo* evaluation of the combined treatment with Vit C + Bnz, the effect of Vit C (13.6 to 81.8 μM) in combination with Bnz (33.3 μM) on trypomastigote stage was evaluated (Table 1).The addition of different concentrations of Vit C (which in the absence of Bnz caused a 4–60% of lysis) to a fixed concentration of Bnz did not increase the trypanocidal activity exerted by Bnz alone.

**Table 1:** Effect of Vit C plus Bnz on *T*. *cruzi* trypomastigotes.

Compounds	Lysis (%)
Control ^(*)^	0
Bnz (33.3 μM)	41,40 ± 3.80
Vit C (13.6 μM)	3.77 ± 1.10
Vit C (13.6 μM) + Bnz (33.3 μM)	35.85 ± 1.50
Vit C (40.9 μM)	26.42 ± 3.50
Vit C (40.9 μM) + Bnz (33.3 μM)	40.40 ± 2.00
Vit C (81.8 μM)	58.94 ± 3.50
Vit C (81.8 μM) + Bnz (33.3 μM)	36.96 ± 4.20

(*) untreated trypomastigotes

### Cytotoxicity assay

**Fig 2:**
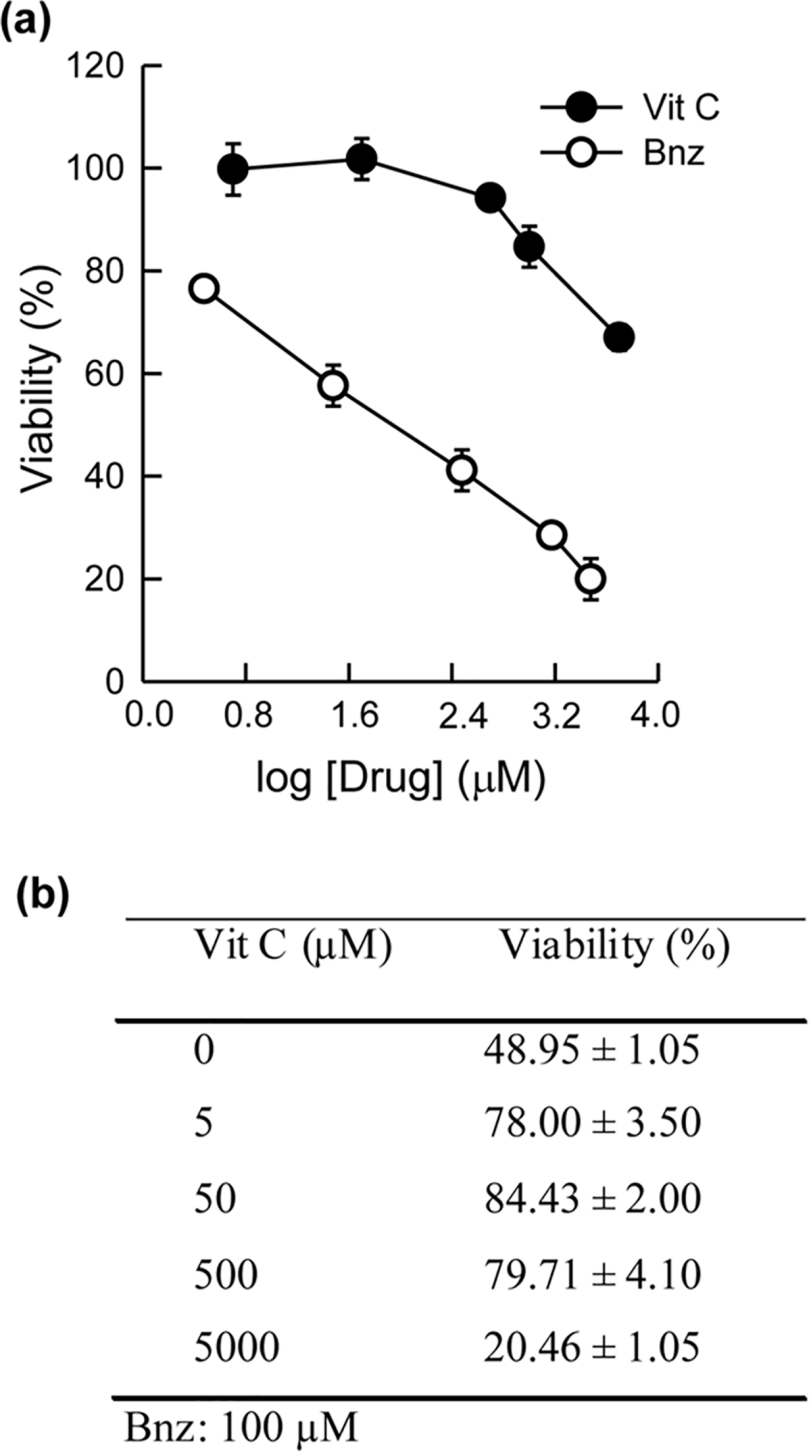
Cytotoxic effect of Vit C and Bnz on Vero cells. **(a).** Cells were cultured for 48 h in the presence of different concentrations of Vit C (5 to 5,000 μM) and Bnz (3 to 3000 μM). Cell viability was determined by the MTT assay (see Methods). **Effect of Vit C on the cytotoxicity of Bnz (b).** In the presence of different concentrations of Vit C (5 to 5000 μM), the cytotoxicity of a fixed concentration of Bnz (100 μM) was evaluated. *Significant differences (p<0.05), when compared to the control, as assessed by the Student’s *t*-test.

Vero cells were treated with different concentrations of Vit C and Bnz (Fig 2A). Vit C did not cause any cytotoxic effect at concentrations of up to 2500 μM. Nevertheless, higher concentrations (5000 μM) proved to exert a cytotoxic effect of 35%. A CC_50_ = 90.9 ± 2.50 μM was obtained for Bnz. The SI is a parameter representing the cytotoxic effects of a drug in mammalian cells, and its antiparasitic activity. SI values were calculated for Vit C and Bnz on all *T*. *cruzi* forms: for epimastigotes, the SI for Bnz was 14.12, whereas for Vit C this value was > 684.93. For trypomastigotes, SI values were 2.74 and > 49.40 for Bnz and Vit C, respectively. For amastigotes, the SI values were 20.10 for Bnz and > 537.63 for Vit C. In order to perform an *in vivo* assessment of the combined treatment with Vit C + Bnz, the effect of Vit C (5 to 5000 μM) on the cytotoxicity exerted by Bnz (100 μM) was assayed (Fig 2B). A significant decrease in the cytotoxicity caused by Bnz was observed in the presence of Vit C at concentrations ranging from 5 to 500 μM. However, this tendency was not observed when 5000 μM of Vit C were added, since cell viability values were the lowest.

### Intracellular redox state assays

**Fig 3:**
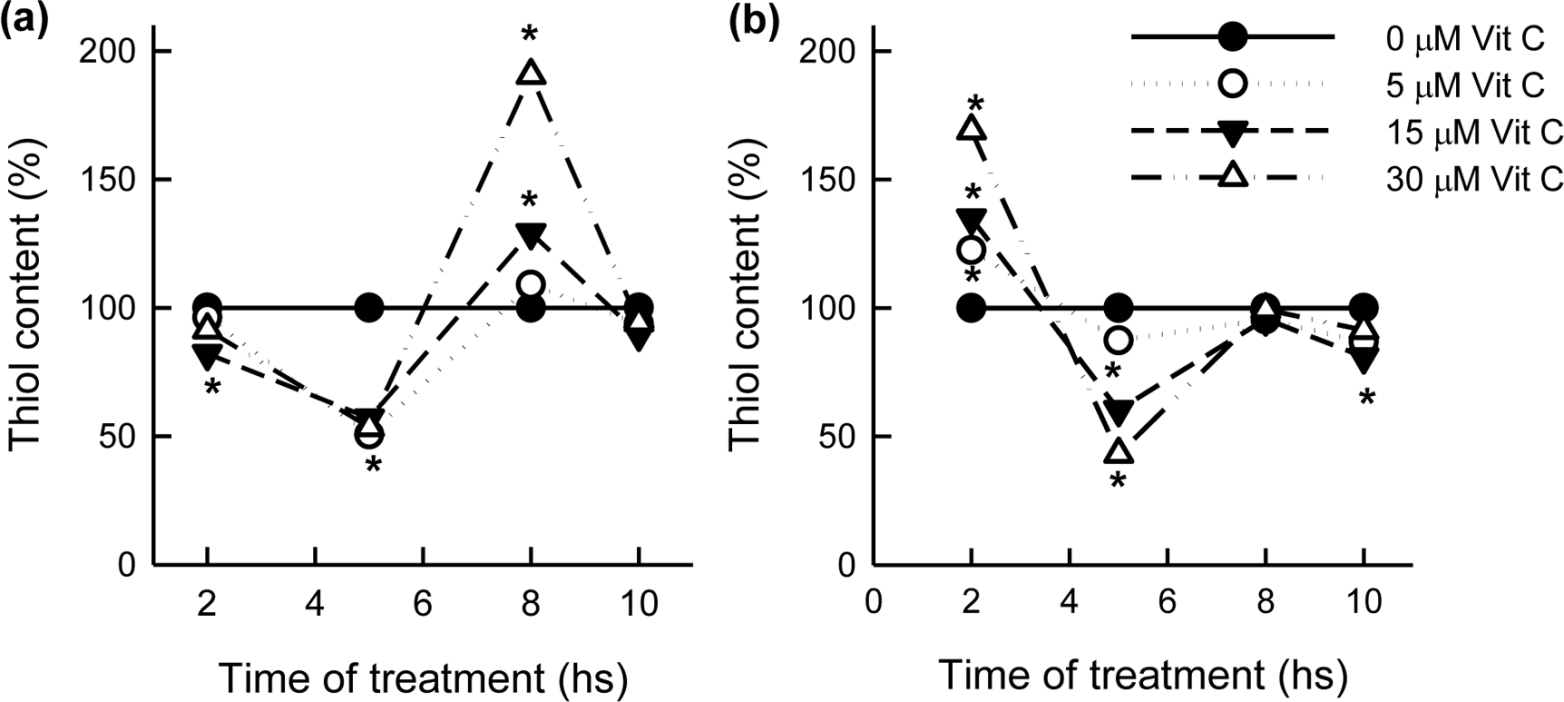
Reduced thiol content during the short-time treatment with different Vit C concentrations. Epimastigotes in culture were treated with Vit C (5, 15 or 30 μM) during 2, 5, 8 and 10 h, in the absence **(a)** or the presence **(b)** of a fixed concentration of Bnz (15μM). The thiol content was considered 100% for parasite cultures incubated without the addition of Vit C in **(a)** parasites alone and in **(b)** parasites treated with Bnz. At different timepoints, parasites were harvested and the thiol content was measured as described in Methods. *Significant differences (p<0.05) when compared to the control, as assessed by the Student’s *t*-test.

Due to its pro-oxidant and/or antioxidant effect, it is postulated that Vit C would induce a redox imbalance leading to changes in both the level of free low molecular weight thiols and the oxidative state inside the parasite. Such changes were evaluated on *T*. *cruzi* epimastigotes treated with different concentrations of Vit C (5, 15 and 30 μM) during short times (2, 5, 8 and 10 h). As shown in Fig 3A, a significant Vit C concentration-independent decrease in the thiol content (~50%) was observed between 2 h and 5 h post-treatment. From 5 h until 8 h, the thiol content increased in a Vit C concentration-dependent manner, reaching maximum values of 191% and 133% (values that were above the control), for 30 μM and 15 μM Vit C, respectively. After reaching this maximum value, the concentration of SH-groups decreased to return to control values after 10 h post-treatment.

This behavior is modified when, to the different Vit C concentrations, a fixed concentration of Bnz (15 μM) is added (Fig 3B). An increase in the content of thiols was observed when the concentrations of Vit C were increased (2 h post-treatment). At 5 h, minimum values of 40, 60 and 85% corresponding to 30, 15 and 5 μM Vit C, respectively were observed. Thiol levels returned to the initial values after 8 h post-treatment and almost no variations were observed between 8 h and 10 h. It is noteworthy that the thiol content in controls (corresponding to different times) observed in presence of Bnz (Fig 3B) is 3 to 5 times lower than those measured in its absence (Fig 3A), which was at all timepoints ~153.52 nmol/mg of protein.

For the treatments described above, no changes in the parasites’ intracellular oxidative stress (evaluated with H_2_DCFDA fluorescent probe) were observed, as compared to controls (S1 Table).

### *In vivo* antiparasitic activity

**Fig 4:**
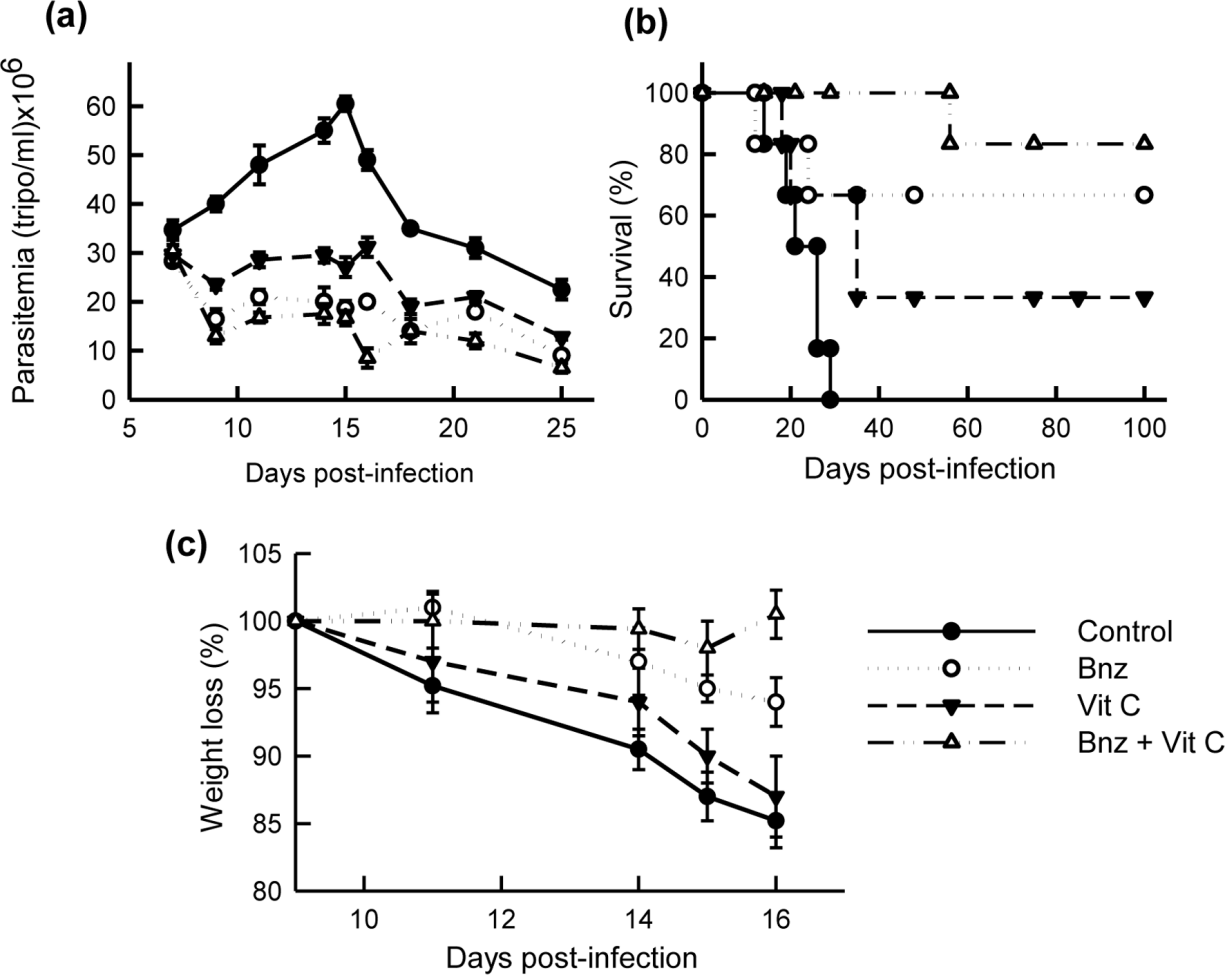
Effect of the treatment with Vit C and Bnz on a murine model of acute Chagas’ disease. C3H mice infected with bloodstream trypomastigotes of *T*. *cruzi* (5 x 10^3^) were treated during days 7 to 11 and 14 to18 post-infection with either Vit C or Bnz only, or with Vit C + Bnz. The control group corresponds to untreated infected animals. Parasitemia levels **(a)** were measured and the survival **(b)** was monitored every day until the end of the treatment. **(c)** The weight loss of each animal was registered between days 9 and 16 post-infection.

The antiparasitic activity of Vit C was measured using a murine model of acute Chagas’ disease. For this experiment, 4 groups of 6 animals each were used, which received Vit C alone or Bnz alone or Vit C plus Bnz or PBS. At the peak of parasitemia (Fig 4A), on day 15, treated groups had lower parasite counts, as compared to the control group (p< 0.01). While a parasitemia value of (60.5 ± 1.5) x 10^6^ parasites/ml was obtained for the control group, the group treated with Vit C alone presented a 55% decrease in such values. Mice treated with Bnz alone or combined with Vit C presented similar parasitemia levels [(18.65 ± 1.5) x 10^6^ and (16.66 ± 1.5) x 10^6^ parasites/ml, respectively]. In terms of area under the parasitemia curves, decreases of 40.45%; 58.65% and 63.79% were observed in mice treated only with Vit C, only with Bnz, or the combination of both, respectively. While in the control group a 100% of mortality was observed between days 14 and 29 post-infection, the group treated with Bnz + Vit C showed 100% survival until the end of the treatment period (Fig 4B). Mice treated with the combination of compounds presented a reduction of weight loss (Fig 4C), which accounts for an improved health status.

## Discussion

Chagas’ disease or American trypanosomiasis, currently treated with nifurtimox and Bnz, requires new and more efficient therapeutic alternatives. Employing the Tulahuén strain, we observed *in vitro* anti-*T*. *cruzi* activity for Vit C on the three parasite forms. This activity is similar that obtained with Bnz (Table 1). In previous studies employing the Y strain of *T*. *cruzi*, the IC_50_ values obtained for Vit C, in the epimastigotes, trypomastigotes and amastigotes, were 550; 9 and 138 times higher the ones obtained herein, respectively [17]. These variations might be caused by two reasons: the different strain of *T*. *cruzi* that was used (Y strain instead of Tulahuén strain) and the range of high concentrations of Vit. C that were tested (0.35 to 2.84 mM instead of 2.5 to 10 μM for epimastigotes, 0.09 to 1.42 mM instead of 0.38 to 380 μM for trypomastigotes and 0.18 to 2.84 mM instead of 0.5 to 40 μM for amastigotes).

It is known that, depending on the concentration, Vit C can act as an antioxidant (at physiological concentrations) or as a pro-oxidant agent (at pharmacological concentrations) [9,10]. As an antioxidant, ascorbate can donate an electron to potentially harmful radicals such as the hydroxyl, alkoxyl, peroxyl, thiol and tocopheroxyl radicals; while being oxidized to the ascorbate radical. To act as a pro-oxidant, the presence of catalytic metal ions (such as Fe, Cu) is required. In the presence of iron, ascorbate is oxidized to the ascorbate radical while it reduces ferric (Fe^3+^) to ferrous (Fe^2+^) iron; Fe^2+^ can rapidly react with O_2_, producing the superoxide radical and Fe^3+^; then superoxide radicals dismutate to H_2_O_2_ and O_2_. Finally, H_2_O_2_ reacts with Fe^2+^ to generate, through the Fenton reaction, Fe^3+^ and the highly oxidant hydroxyl radical. The presence of ascorbate allows the recycling of Fe^3+^ to Fe^2+^, which again catalyzes the formation of highly reactive oxidants from H_2_O_2_ [9,10]. NADH- and NADPH-dependent reductases are responsible for reducing the ascorbate radical back to ascorbate [9,10]. The harmful pro-oxidant effect of Vit C is responsible for cell death and would account for the antiparasitic activity observed on *T*. *cruzi*. The antiparasitic effect of Vit C is also corroborating that, in addition to being synthesized, this compound can be captured from the environment [13] to cause parasite death.

Unlike Bnz, which is cytotoxic, (CC_50_: 90.9 ± 2.50 μM), Vit C showed did not exert a cytotoxic effect, at least at concentrations ≤2,500 μM (Fig 2A). Consequently, the SI value for Vit C is >50, which is considered adequate for trypanocidal drugs [18]. Upon testing Vit C at 5,000 μM, a cytotoxic effect of ~60% was manifested. On the other hand, the cytotoxicity exerted by 100 μM Bnz decreased significantly (from 50 to 20%) in the presence of 5 μM Vit C (Fig 2B).

According to other researchers, the treatment with Vit C alone was not efficient at reducing the parasitemia levels in both the acute and chronic murine model of Chagas’ disease [19,20]. Considering these results and assuming that the secondary effects could be in part related to Bnz cytotoxicity, which significantly decreased in the presence of Vit C, it would be suitable, and taking into account previous works, to implement a combined treatment with Vit C + Bnz. Therefore, the effect of such combination on the viability of trypomastigotes was evaluated in *in vitro* assays (Table 1). Neither additive, synergistic, nor antagonistic effects were observed. The percentage of lysis obtained with the combination of both drugs (Vit C and Bnz) was not different from that obtained with Bnz only. This behavior indicates that the presence of Bnz would abrogate the pro-oxidant effect of Vit C.

Assuming that the level of reduced thiols is as an indicator of the cell redox state; a decrease or an increase in reduced thiol levels could be associated with the pro-oxidant and antioxidant effect of Vit C, respectively. Upon treating epimastigotes with high concentrations of Vit C for short periods of time, (conditions that were previously determined as the most suitable to detect the mode of action [3,4,21], we observed a different behavior as regards thiol levels obtained either in the absence (Fig 3A) or the presence of Bnz (Fig 3B). In the absence of Bnz, the pro-oxidant effect is independent of the concentration of Vit C, and precedes the antioxidant effect; which is Vit. C concentration-dependent and returns the thiol levels to baseline values at the end of the treatment (10 h). When thiol levels are determined after treatment with both Vit C and Bnz (15 μM), first the antioxidant and then the pro-oxidant effect is observed; being both dependent on the vitamin concentration. Metabolomics analyses have identified that the covalent binding of Bnz, or its reduction products, to low molecular weight thiols, as well as to protein thiols, are the main mechanism of action of Bnz on *T*. *cruzi* [22]. This fall in the thiol content caused by Bnz was considered in the control values (0 μM Vit C), therefore it does not affect the results showed in Fig 3B, Considering the depletion of thiol levels produced by Bnz, an antioxidant effect of Vit C, manifested after 2 h after the addition of Bnz, would be expected. The latter effect would contribute to the restoration of baseline thiol levels within the parasite. Results obtained during the first hours of treatment demonstrated a pro-oxidant effect of Vit C in the absence of Bnz, while in the presence of this drug, the antioxidant effect prevails. This finding would explain the lack of pro-oxidant or antitrypanocidal effect of Vit C when added in combination with Bnz on bloodstream trypomastigotes (Table 1).

The treatment with different Vit C concentrations (5 μM to 30 μM) for short periods of time (2 h to 10 h) did not affect the intracellular oxidative stage (S1 Table), thus evidencing the efficacy of the parasite’s defense system to counteract a redox imbalance.

When evaluating the *in vivo* effect of Vit C alone or combined with Bnz, on a murine model of acute Chagas’ disease, a good correlation between the parasitemia levels and the results obtained *in vitro* was observed. Due to the pro-oxidant capacity, mice treated with Vit C had parasitemia levels that were significantly lower than those of control mice, but higher than those obtained with Bnz alone. The combined treatment did not cause a significant decrease in the parasitemia levels, as compared to the levels obtained in animals treated with Bnz alone (in accordance with Table 1). When Vit C is combined with Bnz, its antiparasitic/pro-oxidant effect is not manifested; however, it could be hypothesized that an antioxidant effect would be operating in both parasite and host; protecting the mice from the oxidative stress induced by the infection The latter phenomenon would account for both, the 100% survival and the reduction of weight loss of mice, which accounts for an improved health status. It must be born in mind that the antioxidant effect of Vit C has a stimulatory effect acting on several cell functions of both the innate and adaptive immune system [23]. Therefore, the role of the immune system in animal survival cannot be ruled out (studies currently in progress).

In this paper, the dual effect of Vit C on the parasite was demonstrated. Vit C was proved to have a pro-oxidant effect when administered alone, and an antioxidant effect when used in combination with Bnz. On the host, only the antioxidant effect was evident.

Based on these results, the combined therapy employing Vit C together with Bnz arises as a promising alternative therapy for the treatment of Chagas’ disease. Vit C would also contribute to counteract the serious secondary effects caused by Bnz.

## Supporting information

S1 Fig *In vivo* benznidazole trypanocidal activity. C3H/HeN mice infected with *T*. *cruzi* were treated for 10 days (days 6 to10 and 13 to 17 post-infection) with different concentrations of Bnz. Parasitemia was determined by counting the number of trypomastogotes in a Neubauer chamber. Results shown are representative of three independent experiments.
(TIF)

S1 Table (PDF)
