# Supplementary figures and images for: Anti-parasitic effect of vitamin C alone and in combination with benznidazole against Trypanosoma cruzi
Source: PLoS Negl Trop Dis. 2018 Sep 21;12(9):e0006764. doi: 10.1371/journal.pntd.0006764 (PMC6169970; doi:10.1371/journal.pntd.0006764)

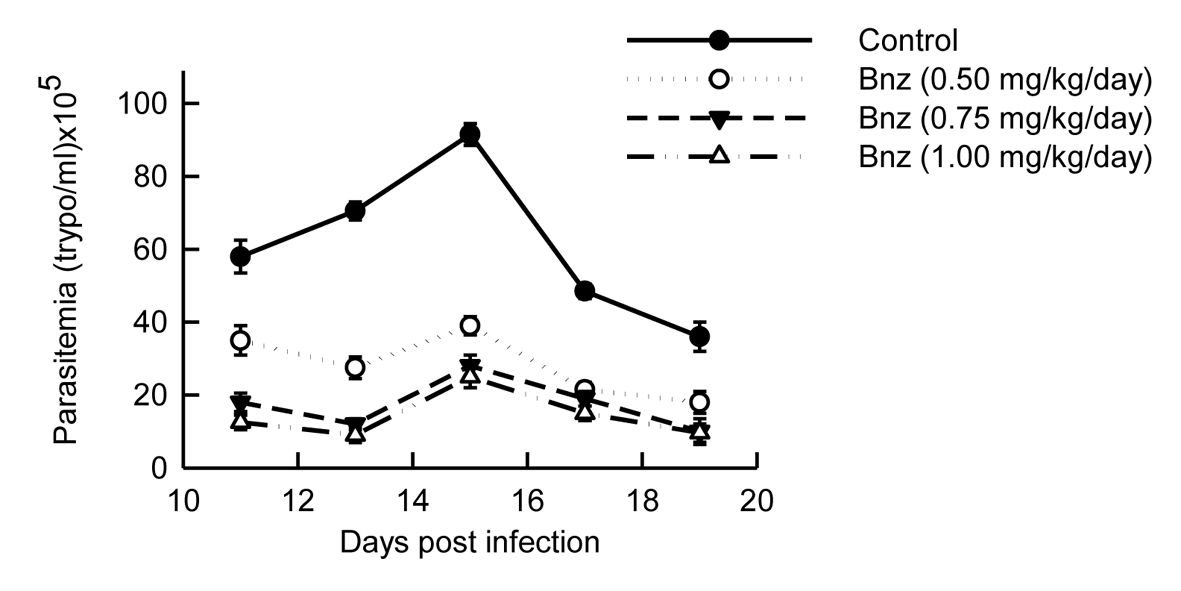

Supplement: S1 Fig — C3H/HeN mice infected with T. cruzi were treated for 10 days (days 6 to10 and 13 to 17 post-infection) with different concentrations of Bnz. Parasitemia was determined by counting the number of trypomastogotes in a Neubauer chamber. Results shown are representative of three independent experiments. (TIF) [file pntd.0006764.s001.tif]
