# Supplementary material for: Anti-parasitic effect of vitamin C alone and in combination with benznidazole against Trypanosoma cruzi
Source: PLoS Negl Trop Dis. 2018 Sep 21;12(9):e0006764. doi: 10.1371/journal.pntd.0006764 (PMC6169970; doi:10.1371/journal.pntd.0006764)
Supplement: S1 Table — (PDF) [file pntd.0006764.s002.pdf]

S1 Table. Intracellular oxidative stress during the treatment with different Vit C concentrations.

Time of treatment (Hours	Control	Treated with Vit C					
		5µM		15 µM		30 µM	
	Gm_c	Gm_t	Gm_t/Gm_c	Gm_t	Gm_t/Gm_c	Gm_t	Gm_t/Gm_c
2	7.39	8.30	1.12	7.40	1.00	8.53	1.15
5	7.93	8.59	1.08	7.81	0.98	8.33	1.05
8	7.03	7.50	1.07	7.89	1.12	7.32	1.04
10	8.20	7.92	0.97	8.80	1.07	7.80	0.95

Gm_t/Gm_c ratios were calculated as indicated in Materials and Methods.
